# Supplementary material for: Epidemiology of Meningococcal Disease in Four South American Countries and Rationale of Vaccination in Adolescents from the Region: Position Paper of the Latin American Society of Pediatric Infectious Diseases (SLIPE)
Source: Vaccines (Basel). 2023 Dec 12;11(12):1841. doi: 10.3390/vaccines11121841 (PMC10748232; doi:10.3390/vaccines11121841)
Supplement: Supplementary file 1 [file vaccines-11-01841-s001.zip › vaccines-2685368-supplementary.pdf]

vaccines

MDPI

Article

Epidemiology of Meningococcal Disease in Four South American Countries and Rationale of Vaccination in Adolescents from the Region: Position Paper of the Latin American Society of Pediatric Infectious Diseases (SLIPE)

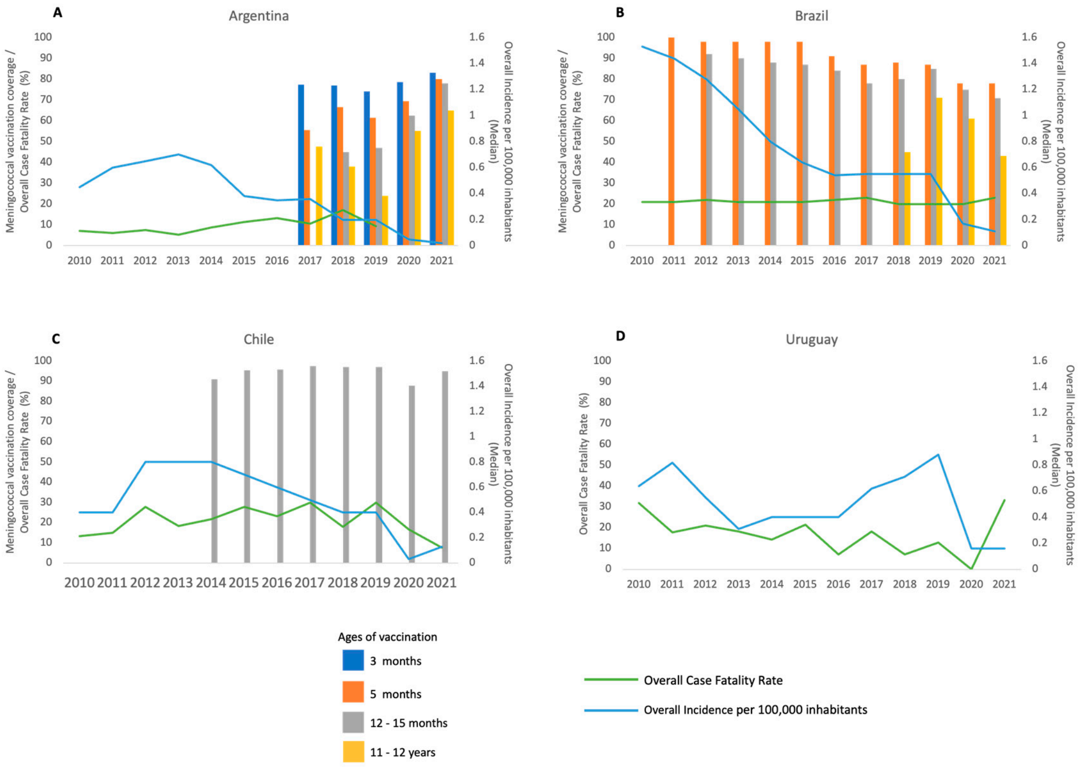

Supplementary Figure S1: Meningococcal vaccination coverage (%), overall incidence and case fatality rates of meningococcal disease in Argentina, Brazil, Chile and Uruguay during the period 2010-2021.
